# Supplementary material for: Mechanism of stepwise electron transfer in six-transmembrane epithelial antigen of the prostate (STEAP) 1 and 2
Source: eLife. 2023 Nov 20;12:RP88299. doi: 10.7554/eLife.88299 (PMC10659578; doi:10.7554/eLife.88299)
Supplement: MDAR checklist [file elife-88299-mdarchecklist1.pdf]

Westbrook Centre
Milton Road
Cambridge CB4 1YG, UK
P 01223 855340
W elifesciences.org
T @elife

Materials Design Analysis Reporting (MDAR)

Title: Mechanism of stepwise electron transfer in six-transmembrane epithelial antigen of the prostate (STEAP) 1 and 2

Authors: Kehan Chen, Lie Wang, Jiemin Shen, Ah-lim Tsai, Ming Zhou, Gang Wu

Materials:

Newly created materials	Indicate where provided: section/figure legend	N/A
The manuscript includes a dedicated "materials availability statement" providing transparent disclosure about availability of newly created materials including details on how materials can be accessed and describing any restrictions on access.	A “materials availability statement” is provided in the end of the manuscript.	

Antibodies	Indicate where provided: section/figure legend	N/A
For commercial reagents, provide supplier name, catalogue number and RRID, if available.	This study does not include any antibody.	

DNA and RNA sequences	Indicate where provided: section/figure legend	N/A
Short novel DNA or RNA including primers, probes: Sequences should be included or deposited in a public repository.	Primer sequences for generating STEAP1 L230G mutant are listed in **Materials and Methods** section.	

Cell materials	Indicate where provided: section/figure legend	N/A
Cell lines: Provide species information, strain. Provide accession number in repository OR supplier name, catalog number, clone number, OR RRID.	This study does not contain cellular study data. All the proteins were expressed in commercial cell lines. The information of the cells used in this study is provided in **Materials and Methods** section.	
Primary cultures: Provide species, strain, sex of origin, genetic modification status.	See above.	

Experimental animals	Indicate where provided: section/figure legend	N/A
Laboratory animals or Model organisms: Provide species, strain, sex, age, genetic modification status. Provide accession number in repository OR supplier name, catalog number, clone number, OR RRID.	No animal (sample) was used in this study.	
Animal observed in or captured from the field: Provide species, sex, and age where possible.	See above.	

Plants and microbes	Indicate where provided: section/figure legend	N/A
Plants: provide species and strain, ecotype and cultivar where relevant, unique accession number if available, and source (including location for collected wild specimens).	This study is not related to plant research.	
Microbes: provide species and strain, unique accession number if available, and source.	This study is not related to microbe research.	

Human research participants	Indicate where provided: section/figure legend) or state if these demographics were not collected	N/A
If collected and within the bounds of privacy constraints report on age, sex, gender and ethnicity for all study participants.	This study does not contain human research.	

Design:

Study protocol	Indicate where provided: section/figure legend	N/A
If the study protocol has been pre-registered, provide DOI. For clinical trials, provide the trial registration number OR cite DOI.		X

Laboratory protocol	Indicate where provided: section/figure legend	N/A
Provide DOI OR other citation details if detailed step-by-step protocols are available.		X

Experimental study design (statistics details) *

**For in vivo studies: State whether and how the following have been done**	**Indicate where provided: section/figure legend. If it could have been done, but was not, write "not done"**	**N/A**
Sample size determination	The study does not contain *in vivo* data.	
Randomisation	See above.	
Blinding	See above.	
Inclusion/exclusion criteria	See above.	

**Sample definition and in-laboratory replication**	**Indicate where provided: section/figure legend**	**N/A**
State number of times the experiment was replicated in the laboratory.	The kinetics measurements were typically conducted ≥ 2 times; the numbers are provided in the figure legends.	
Define whether data describe technical or biological replicates.	The data describes technical replicates.	

**Ethics**	**Indicate where provided: section/submission form**	**N/A**
Studies involving human participants: State details of authority granting ethics approval (IRB or equivalent committee(s), provide reference number for approval.	The study does not involve human participants.	
Studies involving experimental animals: State details of authority granting ethics approval (IRB or equivalent committee(s), provide reference number for approval.	The study does not involve experimental animals.	
Studies involving specimen and field samples: State if relevant permits obtained, provide details of authority approving study; if none were required, explain why.	The study does not involve specimen or field samples.	

**Dual Use Research of Concern (DURC)**	**Indicate where provided: section/submission form**	**N/A**
If study is subject to dual use research of concern regulations, state the authority granting approval and reference number for the regulatory approval.	The study is not subject to dual use research of concern regulations.	

Analysis:

**Attrition**	**Indicate where provided: section/figure legend**	**N/A**
Describe whether exclusion criteria were pre-established. Report if sample or data points were omitted from analysis. If yes, report if this was due to attrition or intentional exclusion and provide justification.	No data point was omitted in kinetic measurements.<br>The criteria for cryo-EM image selection is provided in **Materials and Methods** section.	

**Statistics**	**Indicate where provided: section/figure legend**	**N/A**
Describe statistical tests used and justify choice of tests.	Description of the statistics of Cryo-EM data is provided in **Materials and Methods** section.<br>The error ranges of the kinetic measurements are reported as standard deviations in figure legends and main text, calculated following the standard function.	

**Data availability**	**Indicate where provided: section/submission form**	**N/A**
For newly created and reused datasets, the manuscript includes a data availability statement that provides details for access (or notes restrictions on access).	A data availability statement is provided in the end of the manuscript.	
When newly created datasets are publicly available, provide accession number in repository OR DOI and licensing details where available.	The structure data of STEAP2 are available in the Electron Microscopy Data Bank (access code: EMD-25775): https://www.ebi.ac.uk/emdb/EMD-25775,<br>and the RCSB Protein Data Bank (access code: 7TAI): https://www.rcsb.org/structure/7TAI.<br>The kinetics data of STEAP1 and STEAP2 is available at Dryad: doi:10.5061/dryad.00000008r.	
If reused data is publicly available provide accession number in repository OR DOI, OR URL, OR citation.		X

**Code availability**	**Indicate where provided: section/figure legend**	**N/A**

For any computer code/software/mathematical algorithms essential for replicating the main findings of the study, whether newly generated or re-used, the manuscript includes a data availability statement that provides details for access or notes restrictions.		X
Where newly generated code is publicly available, provide accession number in repository, OR DOI OR URL and licensing details where available. State any restrictions on code availability or accessibility.		X
If reused code is publicly available provide accession number in repository OR DOI OR URL, OR citation.		X

Reporting:

The MDAR framework recommends adoption of discipline-specific guidelines, established and endorsed through community initiatives.

**Adherence to community standards**	**Indicate where provided: section/figure legend**	**N/A**
State if relevant guidelines (e.g., ICMJE, MIBBI, ARRIVE, STRANGE) have been followed, and whether a checklist (e.g., CONSORT, PRISMA, ARRIVE) is provided with the manuscript.		X
